# *In Silico* Affinity Profiling of Neuroactive Polyphenols for Post-Traumatic Calpain Inactivation: A Molecular Docking and Atomistic Simulation Sensitivity Analysis

**DOI:** 10.3390/molecules20010135

**Published:** 2014-12-23

**Authors:** Pradeep Kumar, Yahya E. Choonara, Viness Pillay

**Affiliations:** Wits Advanced Drug Delivery Platform Research Unit, Department of Pharmacy and Pharmacology, School of Therapeutic Sciences, Faculty of Health Sciences, University of the Witwatersrand, Johannesburg, 7 York Road, Parktown 2193, South Africa; E-Mails: pradeep.kumar@wits.ac.za (P.K.); yahya.choonara@wits.ac.za (Y.E.C.)

**Keywords:** spinal cord injury, calpain, calpain inhibitors, polyphenols, curcumin, quercetin, static lattice atomistic simulations, molecular dynamics, molecular docking

## Abstract

Calcium-activated nonlysosomal neutral proteases, calpains, are believed to be early mediators of neuronal damage associated with neuron death and axonal degeneration after traumatic neural injuries. In this study, a library of biologically active small molecular weight calpain inhibitors was used for model validation and inhibition site recognition. Subsequently, two natural neuroactive polyphenols, curcumin and quercetin, were tested for their sensitivity and activity towards calpain’s proteolytic sequence and compared with the known calpain inhibitors via detailed molecular mechanics (MM), molecular dynamics (MD), and docking simulations. The MM and MD energy profiles (SJA6017 < AK275 < AK295 < PD151746 < quercetin < leupeptin < PD150606 < curcumin < ALLN < ALLM < MDL-28170 < calpeptin) and the docking analysis (AK275 < AK295 < PD151746 < ALLN < PD150606 < curcumin < leupeptin < quercetin < calpeptin < SJA6017 < MDL-28170 < ALLM) demonstrated that polyphenols conferred comparable calpain inhibition profiling. The modeling paradigm used in this study provides the first detailed account of corroboration of enzyme inhibition efficacy of calpain inhibitors and the respective calpain–calpain inhibitor molecular complexes’ energetic landscape and in addition stimulates the polyphenol bioactive paradigm for post-SCI intervention with implications reaching to experimental *in vitro*, *in cyto*, and *in vivo* studies.

## 1. Introduction

Traumatic neural injuries, such as brain injury and spinal cord injury (SCI), caused by trauma, ischemia, demyelination, infection, or inflammation, trigger a sequence of events characterized by morphological alterations (axonal degeneration, myelin degradation, vesicularization, and phagocytotic reaction), biochemical variations (extensive relegation of neurofilament protein, microtubule-associated protein 2, myelin basic protein, and proteolipid protein with an increase in intracellular Ca^2+^ levels and tumor necrosis factor-α secretion), and pathophysiological damage (neuronal degeneration and death). In broader terms, the secondary injury mediating tissue damage and destruction prevails over a primary injury’s cellular and molecular pathogenic events, eventually leading to neurological dysfunction and functional paralysis [1]. Several researchers have associated the primary injury outcomes with an elevation in cellular proteases such as calpain, a Ca^2+^-dependent cysteine protease. An increase in intracellular Ca^2+^ levels leads to an increased calpain activity (proteolytic action) in the cystic SCI lesion cavity→degradation of myelin and cytoskeletal proteins + axonal degeneration and myelin vesiculation→destabilization of CNS cellular architecture→neuro tissue destruction [2].

**Figure 1:**
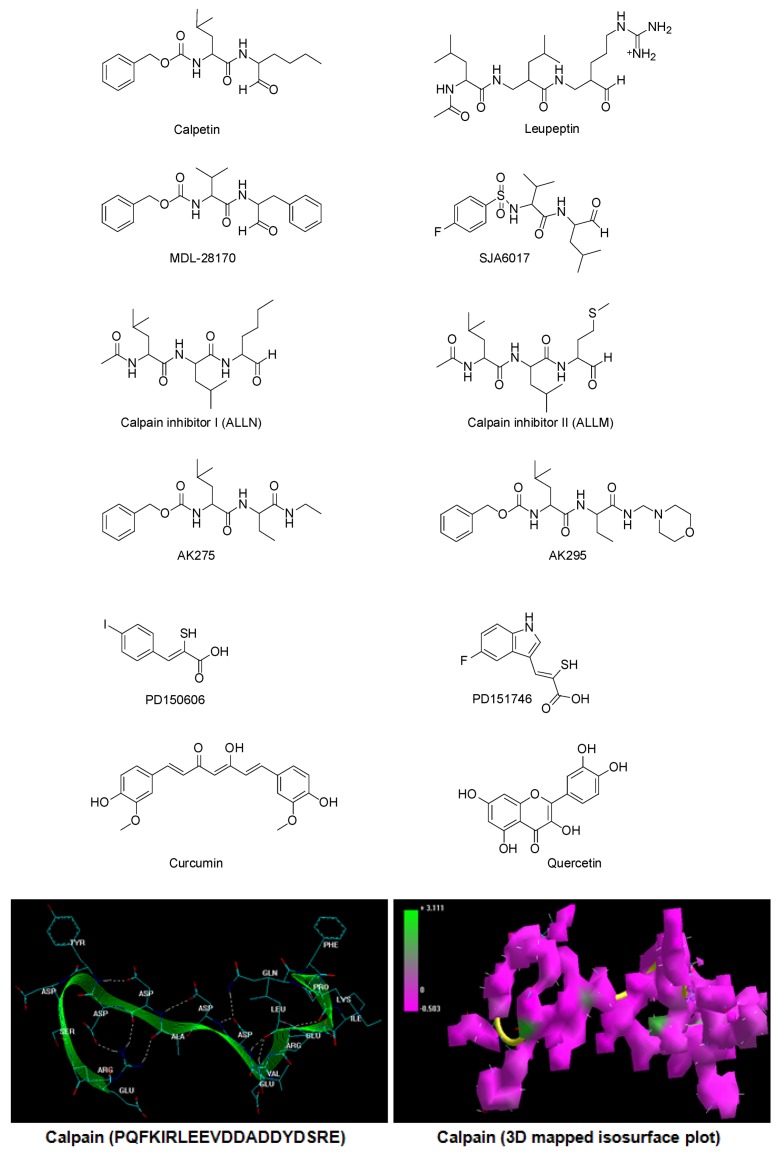
Chemical structures of calpain inhibitors and calpain.

Calpain is profusely expressed in the CNS as two ubiquitous calpain isoforms, μ-calpain and m-calpain, classified based on their activation by μM and mM concentrations of Ca^2+^, respectively. The activity of ubiquitous calpain is regulated by calpastatin, an endogenous protein inhibitor. The activity of calpastatin further depends upon the calpain:calpastatin ratio as an increase in this ratio—overactivation of calpain—leads to degradation of calpastatin, hence restricting its regulatory capacity. Interestingly, both μ-calpain and m-calpain have similar substrate specificity in the CNS and thus there is much debate as to which isoform contributes the most to devastating neural injuries. The intracellular increase in Ca^2+^ after an injury increases calpain activity *in cyto* via an extracellular–intracellular transport mechanism [2]. However, the proteolytic action of calpain on myelin and cytoskeletal proteins cannot be inhibited by therapeutic administration of calpastatin as it lacks cellular permeability [3]. The inability of calpastatin to inhibit calpain activity *in cyto* and the damaging effects of calpain on neuronal architecture makes it a potential therapeutic target to prevent primary and secondary injury cascade. Several research groups around the globe have identified small molecular weight calpain inhibitors capable of cellular permeation and demonstrated their therapeutic potential in various animal models of CNS injuries (brain and spinal cord injuries), neurodegenerative disorders (Alzheimer’s disease, multiple sclerosis, neuronal ischemia, and obsessive-compulsive disorders), and other etiologies (cataract formation, muscular dystrophies, and myocardial infarcts). The calpain inhibitors that have shown immense therapeutic potential in pre-clinical models of traumatic neural injuries are calpain Inhibitor I (ALLN), calpain Inhibitor II (ALLM), AK275, AK295, calpeptin, leupeptin, PD150606, PD151746, MDL-28170, and SJA6017 [4]. Figure 1 illustrates the chemical structures of various synthetic and semisynthetic calpain inhibitors.

Polyphenols or polyhydroxyphenols are natural or synthetic chemical compounds characterized by the presence of multiple phenolic structural units [5]. Natural polyphenolic compounds such as curcumin, quercetin, resvaterol, oleuropein, and epigallocatechin act as antioxidants and are reported for their efficacy in improving the pathophysiological condition caused by traumatic neural injuries. The authors recently hypothesized the combinatorial potential of two specific polyphenols, curcumin (a diferuloylmethane) and quercetin (a flavonoid), in providing neuro-restriction, -repair, -regeneration, -restoration and -reorganization post-SCI [6]. Extending the above hypothesis, this article explores the potential of curcumin and quercetin as inhibitors of calpain activity employing three independent *in silico* molecular modeling techniques: static lattice atomistic simulations (molecular mechanics), molecular dynamics simulations, and molecular docking studies. The molecular attributes of the calpain–curcumin and calpain–quercetin complexes were related to that of well-known calpain inhibitors. For molecular mechanics and dynamics simulations, the 20-mer peptide (PQFKIRLEEVDDADDYDSRE) corresponding to the acidic loop of the calpain molecule—the core sequence known to be the “area of interest” of calpastatin and the inhibition of this calpain Domain III site (the domain containing proteolytic hotspots)—may exert maximal benefits when occupied by small molecules intracellularly in the absence of calpastatin [7,8,9,10,11]. However, to explicate the proteolytic inhibition potential of the tested chemical compounds, the ligands were interacted with the calpain-1 catalytic subunit (RCSB PDB ID: 2R9C) as described by Qian and co-workers, 2008 [12]. This *in silico* analysis provides the foremost detailed molecular interaction analysis of calpain in complexation with cell-permeable calpain inhibitors, with implications reaching to the development of a novel comparative modeling paradigm towards computational testing of the therapeutic potential of protease-inhibitory molecules for future medicinal chemistry applications.

## 2. Results and Discussion

Among the four major mechanisms leading to the initiation of secondary injury after traumatic SCI—(1) compromised blood flow in the spinal cord, (2) intracellular increase in Na^+^, (3) intracellular increase in Ca^++^, and (4) calpain-mediated cytoskeletal proteolytic degradation—calpain activation causes maximum damage through the degradation of cytoskeletal and neurofilamental proteins such as NF68, NF200, microtubule-associated protein 2, and spectrin [13]. With calpain activation beginning as early as 15 min post-SCI, administration of calpain inhibitors may significantly reduce the axonal degeneration by inhibiting the calpain-mediated degradation of cytoskeletal and neurofilamental proteins and may improve the biochemical, functional, and behavioral outcomes.

MM and MD simulations were employed to generate the quantitative and qualitative data pertaining to the bonding and non-bonding energetic transformations and to interpret the electrostatic mapping of the interaction profile of calpain with well-known calpain inhibitors (Table 1 and Table 2). Additionally, comparative correlations between the experimental Ki values and the energetic profiles were obtained. Furthermore, 3D-mapped isosurfaces were employed to generate the electrostatic potential plots. The *in silico* interaction profile of two well-known polyphenolic compounds, curcumin and quercetin, with respect to calpain was investigated using a standard calpain/inhibitor modeling algorithm, and their affinity and effectiveness was ascertained in comparison to known standard calpain inhibitors.

**Table 1:** Inherent energy attributes representing the molecular assemblies modeled using static lattice atomistic simulations in vacuum.

**Molecular Complex**	ΔE ^a^	(*V_∑_*) ^b^	(*V_b_*) ^c^	(*V_θ_*) ^d^	(*V_φ_*) ^e^	(*V_ij_*) ^f^	(*V_hb_*) ^g^	(*V_el_*) ^h^
**μ-Calpain**	−	−374.372	5.323	28.772	31.924	−9.857	−7.086	−423.447
**ALLN**	−	3.059	0.458	2.051	0.978	−0.265	−0.163	0.000
**Calpain–ALLN**	−38.629	−409.942	5.949	31.3229	33.563	−35.922	−7.083	−437.773
**ALLM**	−	2.383	0.436	2.047	0.977	−0.915	−0.161	0.000
**Calpain–ALLM**	−49.433	−421.422	6.065	41.215	37.946	−39.043	−8.419	−459.187
**AK275**	−	2.990	0.449	1.907	0.866	−0.224	−0.008	0.000
**Calpain–AK275**	−19.949	−391.331	5.691	32.126	37.362	−35.689	−8.536	−422.286
**AK295**	−	9.884	0.761	4.971	2.767	1.698	−0.314	0.000
**Calpain-AK295**	−21.328	−385.816	6.096	34.755	37.842	−32.990	−8.609	−422.91
**Calpeptin**	−	5.634	0.440	1.719	0.930	2.647	−0.103	0.000
**Calpain–Calpeptin**	−95.612	−464.350	6.043	33.602	36.133	−30.368	−8.868	−500.893
**Leupeptin**	−	7.255	0.721	3.680	2.918	0.317	−0.382	0.000
**Calpain–Leupeptin**	−27.557	−394.674	5.782	33.716	35.926	−36.288	−7.858	−425.952
**PD150606**	−	29.108	1.768	20.051	0.000	7.382	−0.093	0.000
**Calpain–PD150606**	−36.528	−381.792	5.371	30.470	40.508	−26.740	−6.927	−424.475
**PD151746**	−	24.574	0.432	19.274	3.294	1.669	−0.096	0.000
**Calpain–PD151746**	−21.903	−371.701	5.716	50.131	34.757	−31.057	−7.503	−423.746
**MDL-28170**	−	1.930	0.650	2.029	2.293	−2.692	−0.350	0.000
**Calpain–MDL-28170**	−54.56	−427.002	5.986	31.143	36.182	−43.888	−7.414	−449.013
**SJA6017**	−	29.348	0.491	29.116	1.471	−1.701	−0.0299	0.000
**Calpain–SJA6017**	−17.023	−362.047	6.625	71.023	48.891	−37.210	−9.441	−441.937
**Curcumin**	−	24.900	0.937	7.953	11.153	4.920	−0.064	0.000
**Calpain–Curcumin**	−40.213	−389.685	5.980	35.692	41.923	−37.875	−8.535	−426.87
**Quercetin**	−	13.075	0.792	1.369	3.457	8.183	−0.728	0.000
**Calpain–Quercetin**	−24.825	−386.122	6.231	31.754	35.655	−26.715	−8.998	−424.05

^a^ ΔE_(A/B)_ = E_(A/B)_ − [E_(A)_ + E_(B)_]; ^b^ total steric energy for an optimized structure; ^c^ bond stretching contributions; ^d^ bond angle contributions; ^e^ torsional contribution arising from deviations from optimum dihedral angles; ^f^ van der Waals interactions; ^g^ hydrogen-bond energy function; ^h^ electrostatic energy.

**Table 2:** Energy attributes calculated for the simulated ligand–protein system in a molecular dynamics setup performed in vacuum at 298 K.

Molecular Complex	EPOT ^a^	EKIN ^b^	ETOT ^c^	ΔE ^d^
**μ-Calpain**	−204.279	133.928	−70.351	−
**ALLN**	34.503	27.339	61.914	−
**Calpain–ALLN**	−204.519	160.675	−43.843	−35.406
**ALLM**	33.428	25.364	59.007	−
**Calpain–ALLM**	−225.632	166.344	−59.287	−47.943
**AK275**	31.745	24.642	56.429	−
**Calpain–AK275**	−211.133	177.845	−33.288	−19.366
**AK295**	47.888	25.196	73.085	−
**Calpain–AK295**	−179.914	162.974	−16.940	−19.674
**Calpeptin**	37.271	20.284	57.556	−
**Calpain–Calpeptin**	−267.164	176.158	−91.006	−78.211
**Leupeptin**	45.531	28.330	73.862	−
**Calpain–Leupeptin**	−205.231	167.377	−37.853	−41.364
**PD150606**	37.914	9.389	47.303	−
**Calpain–PD150606**	−205.777	147.914	−57.863	−34.815
**PD151746**	37.452	10.852	48.305	−
**Calpain–PD151746**	−200.149	156.733	−43.415	−21.369
**MDL-28170**	30.058	21.707	51.766	−
**Calpain–MDL**	−233.450	162.201	−71.249	−52.664
**SJA6017**	54.714	20.786	75.501	−
**Calpain–SJA6017**	−167.926	158.503	−9.422	−14.572
**Curcumin**	48.140	19.804	68.070	−
**Calpain–Curcumin**	−193.929	158.120	−35.808	−33.527
**Quercetin**	26.277	16.186	42.464	−
**Calpain–Quercetin**	−210.969	160.157	−50.811	−22.924

^a^ Potential energy component; ^b^ Kinetic energy component; ^c^ Total energy; ^d^ ΔE = E_Total (Ligand-Calpain)_ − [E_Total (Ligand)_ + E_Total (Calpain)_].

The AutoDock program (DockingServer) was employed to perform automated molecular docking simulations. The free energies of binding (ΔE) and inhibition constants (Ki) of the docked compounds were calculated by the Lamarckian Genetic Algorithm and Solis & Wets local search method. The prospective and known inhibitory molecules were successfully docked onto the active site of calpain according to the docking protocol mentioned in the Methods section [14]. Table 3 displays the results obtained from docking simulations.

**Table 3:** Estimated free energies of binding, constituent energies, calculated inhibition constants [Ki (calculated)], and interaction surface of the studied inhibitors after docking into the proteolytic site of calpain.

Molecular Complex	Est. Free Energy of Binding (kcal/mol)	Est. Inhibition Constant, Ki	vdW ^a^ + Hbond ^b^ + Desolv Energy ^c^ (kcal/mol)	Electrostatic Energy (kcal/mol)	Total Intermol. Energy ^d^ (kcal/mol)	Interact Surface
Calpain–ALLN	−3.59	2.32 mM	−6.61	−0.03	−6.64	724.689
Calpain–ALLM	−5.33	123.79 μM	−6.39	−0.03	−6.42	730.628
Calpain–AK275	−2.99	6.48 mM	−5.33	0.00	−5.33	680.763
Calpain–AK295	−3.05	5.81 mM	−6.70	−0.10	−6.81	807.051
Calpain–Calpeptin	−4.88	263.03 μM	−7.28	−0.05	−7.34	717.018
Calpain–Leupeptin	−4.19	842.98 μM	−6.02	−1.41	−7.42	858.214
Calpain–PD150606	−3.97	1.23 mM	−4.86	−0.06	−4.92	386.704
Calpain–PD151746	−3.54	2.53 mM	−4.63	+0.50	−4.14	441.371
Calpain–MDL-28170	−5.03	204.69 μM	−7.07	−0.07	−7.14	704.099
Calpain–SJA6017	−4.90	254.11 μM	−6.59	−0.09	−6.68	656.198
Calpain–Curcumin	−4.11	978.21 μM	−5.52	+0.04	−5.49	629.613
Calpain–Quercetin	−4.69	365.57 μM	−4.76	−0.14	−4.90	494.757

^a^ van der Waals energy; ^b^ Hydrogen bonding; ^c^ Desolvation energy; ^d^ Total intermolecular energy.

### 2.1. Tripeptidyl Aldehydes

**Figure 2:**
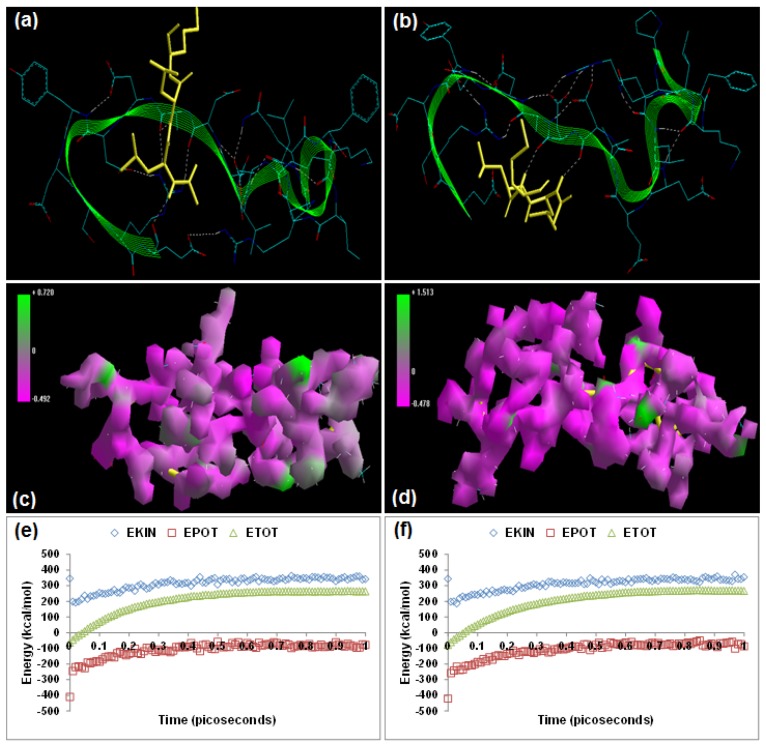

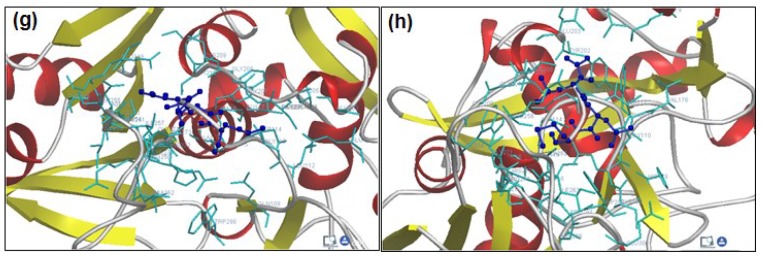
Visualization of the geometrical preferences of the calpain molecule (green secondary structure) in complexation with ligand molecules (yellow tube rendering). (**a**) Calpain inhibitor I (ALLN) and (**b**) calpain inhibitor II (ALLM) after molecular mechanics simulations. 3D-mapped isosurface plot representing the electrostatic potential for (**c**) calpain–ALLN and (**d**) calpain–ALLM. The kinetic energy (EKIN), potential energy (EPOT), and total energy (ETOT) values for (**e**) calpain–ALLN and (**f**) calpain–ALLM, calculated by molecular dynamic simulations in vacuum. The most favorable poses of (**g**) ALLN and (**h**) ALLM within calpain were obtained via molecular docking.

Calpain Inhibitor I (ALLN) [(2*R*)-2-[(2*S*)-2-acetamido-4-methylpentanamido]-4-methyl-N-[(2*S*)-1-oxohexan-2-yl]pentanamide] and calpain Inhibitor II (ALLM) [(2*R*)-2-[(2*S*)-2-acetamido-4-methylpentanamido]-4-methyl-N-[(2*S*)-4-(methylsulfanyl)-1-oxobutan-2-yl] pentanamide] have been reported to prevent proteolysis and neuronal apoptosis when administered intravenously after spinal cord injury [15,16]. Energetic calculations following MM simulations revealed that ALLM (ΔE_Total_ = −49.433 kcal/mol) conferred better interaction and complexation with calpain than ALLN (ΔE_Total_ = −38.629 kcal/mol), which is in agreement with the reported inhibitor profile of ALLM [calpain I (Ki = 120 nM) and calpain II (Ki = 230 nM)] and ALLN [calpain I (Ki = 190 nM), calpain II (Ki = 220 nM)] ([17], Table 1). The presence of methylsulfanyl functionality in ALLM significantly contributed to the magnitude of intrinsic energy components. The electrostatic component, major contributor to the calpain–calpain inhibitor geometrical stabilization, of the calpain–ALLM (−459.187 kcal/mol) was more stabilized than that of calpain–ALLN (−437.773 kcal/mol). The 3D mapped isosurface plot of calpain–ALLN (−0.492 kcal/mol < V < +0.720 kcal/mol) depicted overall strong and weak, minor and major, negative and positive potentials dispersed throughout the isosurface. Conversely, ALLM complexed with calpain (−0.478 kcal/mol < V < +1.513 kcal/mol) via the formation of a strong positive minor electrostatic surface and a major electrostatic surface with strong negative potential, proving a calpain-amenable geometrical distribution of electrostatic potential across the ligand–protein interface. The methylsulfanyl group significantly altered the geometrical orientation of calpain via torsional constraints leading to the destabilization of all three bonding energies—bond length, bond angle, and dihedral angle—which was compensated for by the electrostatic stabilization and hence demonstrated a better fit in the proteolytic cavity (Figure 2). Correspondingly, the MD simulations demonstrated better energetic stabilization in the case of calpain–ALLM (ΔE_Total_ = −47.943 kcal/mol) than calpain–ALLN (ΔE_Total_ = −35.406 kcal/mol) with both component kinetic and potential energies contributing to final geometrical and energetic equilibrium (Table 2).

Calpain inhibitors I and II displayed –ve free energy of binding with the proteolytic site of calpain for all binding modes tested (Table 3). As observed in MM and MD simulations, ALLM (ΔE_binding_ = −5.33 kcal/mol) demonstrated better inhibition of calpain with a Ki value of 123.79 μM as compared to ALLN (ΔE_binding_ = −3.59 kcal/mol), which showed a Ki value of 2.32 mM. Although the total intermolecular energy component was more stabilized in the case of the calpain–ALLN complex, the calpain–ALLM complex demonstrated higher interaction surface and hence a better geometrical fit. The binding pocket of calpain–ALLN was lined by GLU72, GLN109, CYS115, TRP116, THR210, SER206, SER251, ASN253, ILE254, ARG258, ALA262, VAL269, GLY271, HIS272, ALA273, and TRP298, with hydrogen bonding interactions (GLY271 and CYS115), polar bonds (HIS272, GLN109, ASN253, ARG258, and TRP298), and hydrophobic involvements (HIS272, VAL269, ALA262, CYS115, TRP116, ILE254, ALA273, and TRP298) contributing to the binding energy (Figure 2). The hydrogen bonding length in the calpain–ALLN molecular complex ranged between 3.08 and3.42. In the case of calpain–ALLM, the ligand–protein binding included GLU72, GLN109, LEU112, CYS115, TRP116, SER206, GLY208, SER251, ASN253, ILE254, ARG258, ALA262, VAL269, GLY271, HIS272, ALA273, and TRP298, with interaction arising from hydrogen bonding (GLY271 and CYS115), polar bonds (GLN109, HIS272, GLU72, SER206, ARG258, and TRP298), and hydrophobic deliberations (CYS115, ILE254, VAL269, HIS272, TRP298, ALA262, TRP116, and ALA273) (Figure 2). The hydrogen bond length in the case of calpain–ALLM ranged between 2.92 and 3.50, displaying both long and short range H-bonding interactions. Hence, the better inhibitory potency of calpain–ALLM could be attributed to (1) better hydrogen bonding, (2) a larger binding pocket, (3) favorable torsional constraints, and (4) balanced electrostatic mapping—in corroboration with the binding energy and inhibition constant values.

### 2.2. Dipeptidyl α-Keto Amides

**Figure 3:**
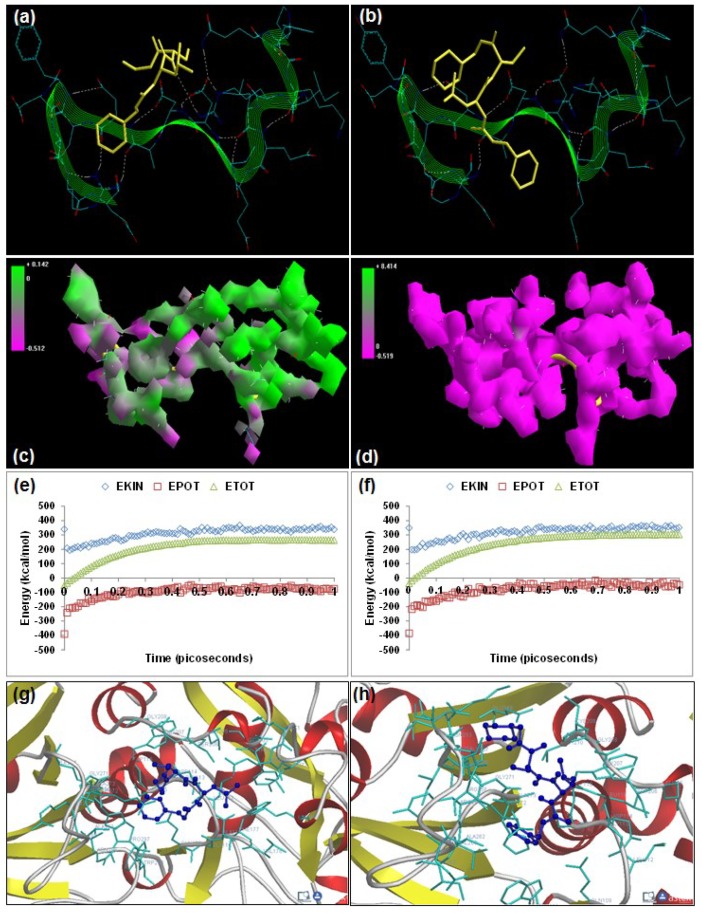
Visualization of the geometrical preferences of the calpain molecule (green secondary structure) in complexation with ligand molecules (yellow tube rendering). (**a**) AK275 and (**b**) AK295 after molecular mechanics simulations. 3D-mapped isosurface plot representing the electrostatic potential for (**c**) calpain–AK275 and (**d**) calpain–AK295. The kinetic energy (EKIN), potential energy (EPOT), and total energy (ETOT) values for (**e**) calpain–AK275 and (**f**) calpain–AK295 calculated by molecular dynamic simulations in vacuum. The most favorable poses of (**g**) AK275 and (**h**) AK295 within calpain were obtained via molecular docking.

The oxoamide inhibitors AK275 (benzyl N-[(1*S*)-1-{[(1*S*)-1-(ethylcarbamoyl)propyl] carbamoyl}-3-methylbutyl]carbamate) and AK295 (benzyl N-[(1*S*)-3-methyl-1-{[(1*S*)-1-[(morpholin-4-ylmethyl)carbamoyl]propyl]carbamoyl}butyl]carbamate) are potent, highly specific, and permeable neuroentities differing in their aqueous solubility characteristics, with AK295 being more soluble than AK275, and are known to inhibit calpain-induced degeneration *in vivo* as well as inhibition of *in vitro* proteolysis of cytoskeletal proteins [18,19]. Energetic calculations following MM simulations revealed that AK295 (ΔE_Total_ = −21.328 kcal/mol) conferred better interaction and complexation profile with calpain than AK275 (ΔE_Total_ = −19.949 kcal/mol), which corroborated the reported experimental inhibition capability of AK295 [inhibitor of calpain I (Ki = 0.150 μM), calpain II (Ki = 0.041 μM)] as compared to AK275 [calpain I (Ki = 0.25 μM) and calpain II (Ki = 0.21 μM)] [12], Table 1]. The presence of morpholine functionality in AK295 contributed significantly to the bond angle, torsional strain, van der Waals non-bonding, and H-bonding energy components intrinsic to dipeptidyl α-keto amides. The influence of this functionality was additionally reflected in the final geometrically optimized molecular complexes, wherein calpain–AK295 was energetically stabilized via the bond angle energy component (V_θ_) and electrostatic forces (V_el_). However, the van der Waals forces (V_ij_) component of calpain–AK295 (−8.609 kcal/mol) was equally stabilized to that of calpain–AK275 (−8.536 kcal/mol). Surprisingly, both AK275 and AK295 destabilized the non-bonding electrostatic energy of calpain (−423.447 kcal/mol). The 3D-mapped isosurface plots of calpain–AK275 (−0.512 kcal/mol < V < +0.142 kcal/mol) and calpain–AK295 (−0.519 kcal/mol < V < +8.414 kcal/mol) depicted completely opposite electrostatic surfaces with predominantly strong positive major and strong negative major electrostatic surfaces, respectively, proving the complementarity of AK295 towards calpain’s electrostatic map, and hence maintained the geometrical distribution of electrostatic potential across the ligand–protein interface. Furthermore, the 3D mapping verifies the significant contribution of morpholine moiety towards the electrostatic component of the enzyme/inhibitor complex. However, the morpholino-functionalization of AK275 to obtain AK295 decreased the H-bonding between the ligand–protein as evident from Figure 3 and from the MM energy paradigms shown in Table 1. Therefore, the molecular optimization in calpain–AK295 can be attributed to the altered geometrical orientation of calpain via torsional constraints leading to the stabilization of dihedral and hydrophobic components, showing a better fit in the active site of calpain (Figure 3). Correspondingly, the MD simulations demonstrated better energetic stabilization in the case of demonstrated calpain–AK295 (ΔE_Total_ = −19.674 kcal/mol) than calpain–AK275 (ΔE_Total_ = −19.366 kcal/mol), with both component kinetic and potential energies contributing to final geometrical and energetic equilibrium (Table 2).

AK275 and AK295 displayed –ve free energy of binding with the proteolytic site of calpain for all binding modes tested (Table 3). As observed in MM and MD simulations, AK295 (ΔE_binding_ = −3.05 kcal/mol) demonstrated better inhibition of calpain, with a Ki value of 5.81 mM as compared to AK275 (ΔE_binding_ = −2.99 kcal/mol), which showed a Ki value of 6.48 mM. All the intermolecular energy components—van der Waals + H-bond + desolvation + electrostatic—were more stabilized in the case of the calpain–AK295 complex, and the larger interaction surface produced a better geometrical fit. The binding pocket of calpain–AK275 was formed by GLU72, LYS79, GLN109, CYS115, THR210, SER206, SER251, ILE254, ARG258, ALA262, VAL269, ARG270, GLY271, HIS272, ALA273, TRP298, 347LYS, and 349GLU, with hydrogen bonding interactions (CYS115, ARG270, GLY271, HIS272, and ILE254), polar bonds (SER251, HIS272, GLN109, and ARG258), and hydrophobic involvements (CYS115, HIS272, TRP298, VAL269, ILE254, ALA262, and ALA273) contributing to the binding energy (Figure 3). The hydrogen bonding length in calpain–AK275 molecular complex ranged between 2.86 and 3.82. In the case of calpain–AK295, the ligand–protein binding included GLU72, LYS79, GLN109, CYS115, TRP116, SER206, SER251, ASN253, ILE254, ARG258, GLU261, ALA262, 263ILE, VAL269, GLY271, HIS272, and TRP298, with interaction arising from hydrogen bonding (CYS115, SER206, SER251, GLY271, HIS272, and ILE254), polar bonds (GLU72, ASN253, GLN109, HIS272, and TRP298), hydrophobic deliberations (ILE254, VAL269, ALA262, TRP116, CYS115, TRP116, TRP298, HIS272, and ILE263), π–π bonding (HIS272 and TRP298), and cation–π interactions (TRP298) (Figure 3). The hydrogen bond length in the case of calpain–AK295 ranged between 2.63 and 3.58, displaying shorter H-bonding interactions than calpain–AK275. Hence, calpain–AK295 showed stronger hydrogen bonding as well as a larger binding pocket, in line with the binding energy and inhibition constant values. The final geometrically optimized complexes of AK275 and AK295 with calpain after MM, MD, and docking simulations revealed very close finalized binding energy values. However, the introduction of a morpholine group in AK295 rendered more functionality to the molecule, leading to π–π and cation–π interactions with calpain and hence contributing to “efficient and selective inhibition” of calpain’s proteolytic action.

### 2.3. Dipeptidyl Aldehydes

**Figure 4:**
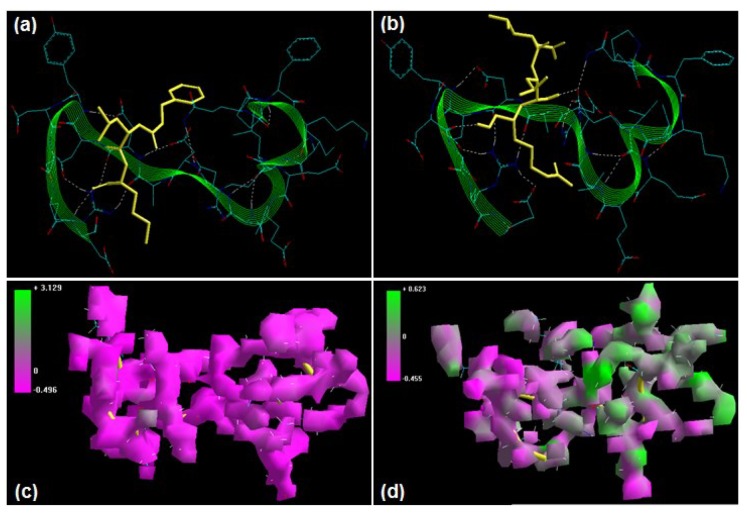

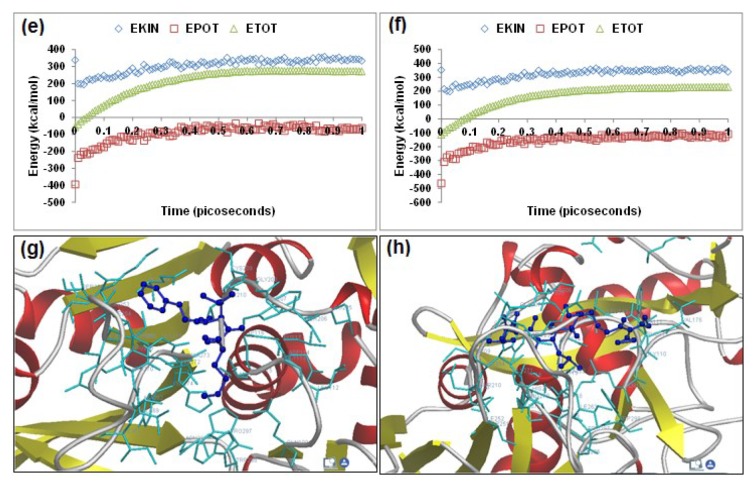
Visualization of the geometrical preferences of the calpain molecule (green secondary structure) in complexation with ligand molecules (yellow tube rendering). (**a**) Calpeptin and (**b**) leupeptin after molecular mechanics simulations. 3D-mapped isosurface plot representing the electrostatic potential for (**c**) calpain–calpeptin and (**d**) calpain–leupeptin. The kinetic energy (EKIN), potential energy (EPOT), and total energy (ETOT) values for (**e**) calpain–calpeptin and (**f**) calpain–leupeptin calculated by molecular dynamic simulations in vacuum. The most favorable poses of (**g**) calpeptin and (**h**) leupeptin within calpain were obtained via molecular docking.

Prototypical peptidyl aldehyde calpain inhibitors—calpeptin (benzyl N-[(1*S*)-3-methyl-1-{[(2*R*)-1-oxohexan-2-yl]carbamoyl}butyl]carbamate) and leupeptin ((2*S*)-N-[(1*S*,2*S*)-1-{[(2*R*)-5-Carbamimidamido-1-hydroxypentan-2-yl]amino}-1-hydroxy-4-methylpentan-2-yl]-2-acetamido -4-methylpentanamide)—differ in terms of chemical structure, permeability, charge, and selectivity, with calpeptin containing benzyl functionality and being cell permeable, less aqueous soluble, and higher in selectivity, while leupeptin is positively charged due to the presence of guanidinium group, water soluble, poorly cell permeable, and less selective towards calpain inhibition. Calpeptin and leupeptin are among the first calpain inhibitors to be employed to demonstrate “the involvement of calpain in apoptotic death of rat glial and neuronal cells” [20,21]. Energetic calculations following MM simulations revealed that calpeptin (ΔE_Total_ = −95.612 kcal/mol) conferred better interaction and complexation with calpain than leupeptin (ΔE_Total_ = −27.557 kcal/mol), which is in line with the reported significantly different inhibition profiles of calpeptin [calpain I (Ki = 0.067 μM) and calpain II (Ki = 0.062 μM)] and leupeptin [calpain I (Ki = 0.27 μM), calpain II (Ki = 0.38 μM)] ([19], Table 1). The presence of benzyl functionality in calpeptin contributed immensely to the energy components intrinsic to these dipeptidyl aldehydes. The electrostatic component, the major contributor to the calpain–calpain inhibitor geometrical stabilization, of the calpain–calpeptin (−500.893 kcal/mol) was more stabilized than that of calpain–leupeptin (−425.952 kcal/mol). In fact, leupeptin destabilized the electrostatic architecture of calpain due the presence of a positively charged guanidinium group. The 3D-mapped isosurface plot of calpain–calpeptin (−0.496 kcal/mol < V < +3.129 kcal/mol), like calpain–ALLM, depicted a strong positive minor electrostatic surface and a major electrostatic surface with strong negative potential, presenting a balanced electrostatic geometry capable of stabilizing the molecular conformation. However, the calpain–leupeptin complex (−0.455 kcal/mol < V < +0.623 kcal/mol) was characterized by strong and weak, minor and major, negative and positive potentials, showing unequal distribution of charge. The benzyl group, however, insignificantly altered the torsional and bonding energies. On the other hand, the charged guanidinium functionality in leupeptin contributed (although not significantly) towards the van der Waals and H-bonding interactions (Figure 4). Correspondingly, the MD simulations demonstrated better energetic stabilization in the case of demonstrated calpain–calpeptin (ΔE_Total_ = −78.211 kcal/mol) than calpain–leupeptin (ΔE_Total_ = −41.364 kcal/mol), with both component kinetic and potential energies contributing to final geometrical and energetic equilibrium (Table 2).

Calpeptin and leupeptin displayed –ve free energy of binding with the proteolytic site of calpain for all binding modes tested (Table 3). As observed in MM and MD simulations, calpeptin (ΔE_binding_ = −4.88 kcal/mol) demonstrated better inhibition of calpain with a Ki value of 263.03 μM as compared to leupeptin (ΔE_binding_ = −4.19 kcal/mol), which showed a Ki value of 842.98 μM. The major intermolecular energy components—van der Waals + H-bond + desolvation—were more stabilized in the case of the calpain–calpeptin complex. However, the calpain–leupeptin complex demonstrated higher interaction surface, which may be due to a larger molecular size and hence may not be able to provide a preferable geometrical fit. The binding pocket of calpain–calpeptin was lined by GLU72, GLN109, CYS115, TRP116, THR210, SER251, ASN253, ILE254, ARG258, ALA262, VAL269, GLY271, HIS272, ALA273, TRP298, and GLU349, with hydrogen bonding interactions (GLY271 and CYS115), polar bonds (THR210, HIS272, ARG258, GLN109, and TRP298), hydrophobic involvements (ALA111, CYS115, TRP116, HIS272, TRP298, VAL269, ILE254, ALA273, and ALA262), and cation–π interaction (TRP298) contributing to the binding energy (Figure 4). The hydrogen bonding length in the calpain–leupeptin molecular complex ranged between 2.63 and 3.25. In the case of calpain–leupeptin, the ligand–protein binding included GLU72, LYS79, GLN109, GLY110, ALA111, LEU112, CYS115, TRP116, SER206, GLY208, CYS209, THR210, SER251, ASN253, ILE254, ARG258, VAL269, GLY271, HIS272, ALA273, TRP298, LYS347, and GLU349, with interaction arising from hydrogen bonding (GLU72, CYS115, GLY208, GLY110, THR210, ASN253, GLU349, and GLY271), polar bonds (GLU72, GLN109, TRP116, LYS347, GLU349, THR210, and SER251), hydrophobic deliberations (LEU112, CYS209, CYS115, TRP116, ILE254, VAL269, HIS272, ALA273, and TRP298) and cation–π interaction (TRP116) (Figure 4). The hydrogen bond length in the case of calpain–leupeptin ranged between 2.52 and 3.36, displaying both long- and short range H-bonding interactions.

The hydrogen bonding and large binding pocket in the case of calpain–leupeptin failed to corroborate the binding energy and inhibition constant values, revealing that an appropriate molecular size and charge are key to inhibition of calpain’s proteolytic site.

### 2.4. α-Mercapto Acrylic Acids

**Figure 5:**
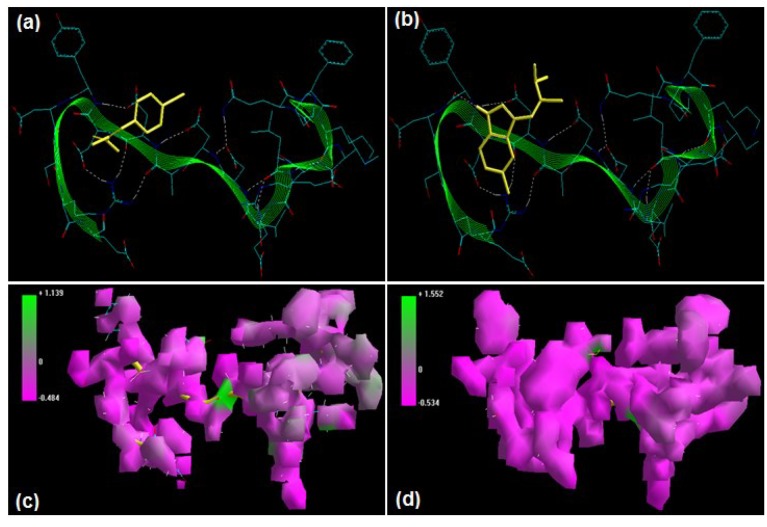

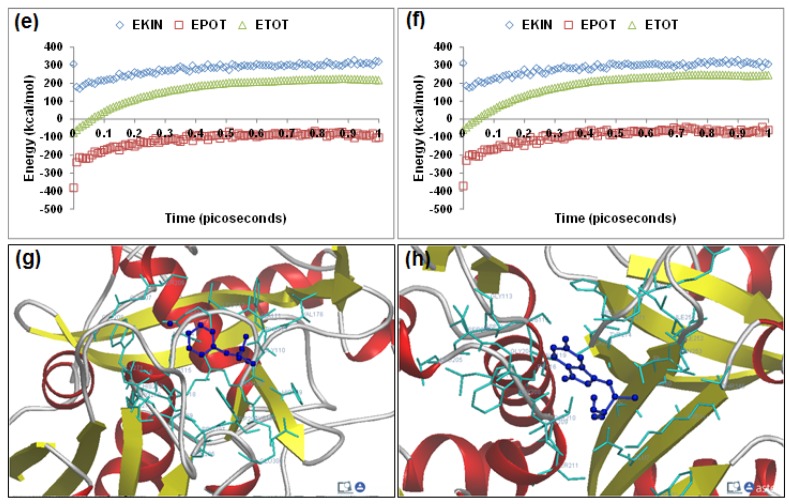
Visualization of the geometrical preferences of the calpain molecule (green secondary structure) in complexation with ligand molecules (yellow tube rendering), (**a**) PD150606 and (**b**) PD151746 after molecular mechanics simulations. 3D-mapped isosurface plot representing the electrostatic potential for (**c**) calpain–PD150606 and (**d**) calpain–PD151746. The kinetic energy (EKIN), potential energy (EPOT), and total energy (ETOT) values for (**e**) calpain–PD150606 and (**f**) calpain–PD151746 calculated by molecular dynamic simulations in vacuum. The most favorable poses of (**g**) PD150606 and (**h**) PD151746 within calpain were obtained via molecular docking.

PD150606 (3-(4-iodophenyl)-2-mercapto-(Z)-2-propenoic acid) and PD151746 ([(1E)-1-(5-fluoro-1H-indol-3-yl)-3,3-dihydroxyprop-1-en-2-yl]sulfanide) represent the most potent α-mercapto acrylic acids, characterized by higher cell permeability, small molecular weight, and the presence of a halogen such as fluorine or iodine [22,23,24]. Energetic calculations following MM simulations revealed that PD150606 (ΔE_Total_ = −36.528 kcal/mol) conferred better interaction and complexation with calpain than PD151746 (ΔE_Total_ = −21.903 kcal/mol), which supported the reported experimental inhibition capability of PD150606 [calpain I (Ki = 0.21 μM) and calpain II (Ki = 0.37 μM)] as compared to PD151746 [calpain I (Ki = 0.26 μM) and calpain II (Ki = 5.3 μM)] ([17], Table 1). The presence of iodo functionality in PD150606 contributed significantly to the bond length, bond angle, and electrostatic energy stabilization in calpain–PD150606, while the fluoro functionality in PD151746 stabilized the torsional energy, van der Waals interaction, and H-bonding energy components in the calpain–PD151746 complex. Geometrically, the bond angle energy component contributed maximally to the final molecular optimization in calpain–PD150606. However, the electrostatic component in case of calpain–PD150606 was equivalent to that of calpain–PD151746. The reduced inhibitory potential of PD151746 can be attributed to the destabilization of bond angle energy of calpain from 28.772 kcal/mol to 50.131 kcal/mol, as compared to 30.470 kcal/mol in the case of calpain–PD150606. The 3D-mapped isosurface plots of calpain–PD150606 (−0.484 kcal/mol < V < +1.139 cal/mol) and calpain–PD151746 (−0.534 kcal/mol < V < +1.552 kcal/mol) depicted strong major negative electrostatic potential surfaces maintaining the equivalent geometrical distribution of electrostatic potential across the ligand–protein interface (Figure 5). Furthermore, 3D mapping verifies the equivalent contribution of halogen moieties in PD150606 and PD151746 towards the finalized electrostatic component of ligand–protein complexes. Therefore, the molecular optimization in calpain–PD150606 can be attributed to the altered the geometrical orientation of calpain via bond angle changes and non-bonding components leading to a better fit in the active site of calpain. Correspondingly, the MD simulations demonstrated better energetic stabilization in case of demonstrated calpain–PD150606 (ΔE_Total_ = −34.815 kcal/mol) than calpain–PD151746 (ΔE_Total_ = −21.369 kcal/mol) with both component kinetic and potential energies contributing to final geometrical and energetic equilibrium (Table 2).

PD150606 and PD151746 displayed –ve free energy of binding with the proteolytic site of calpain for all binding modes tested (Table 3). As observed in MM and MD simulations, PD150606 (ΔE_binding_ = −3.97 kcal/mol) demonstrated better inhibition of calpain, with a Ki value of 1.23 mM as compared to PD151746 (ΔE_binding_ = −3.54 kcal/mol), which showed a Ki value of 2.53 mM. All intermolecular energy components—van der Waals + H-bond + desolvation + electrostatic—were more stabilized in the case of the calpain–PD150606 complex, which also had a larger interaction surface, producing a better geometrical fit. The binding pocket of calpain–PD150606 was lined by GLN109, GLY113, CYS115, TRP116, SER251, ILE254, ALA262, VAL269, ARG270, GLY271, HIS272, TRP298, LYS347, and 349GLU, with hydrogen bonding interactions (SER251 and TRP298), halogen bonds (GLY208, GLY209GLY113, ARG270, and GLY271), polar bonds (GLN109, SER251, HIS272, LYS347, and GLU349), hydrophobic involvements (TRP208, ALA262, HIS272, and VAL269), and π–π bonds (HIS272 and TRP298) contributing to the binding energy (Figure 5). The hydrogen bonding length in the calpain–PD150606 molecular complex was 3.40. In the case of calpain–PD151746, the ligand–protein binding included GLU72, LEU73, 79LYS, GLN109, LEU112, GLY113, CYS115, TRP116, GLU203, SER206, THR210, SER251, ILE252, ASN253, ILE254, ILE257, ARG258, ALA262, VAL269, ARG270, GLY271, HIS272, ALA273, TRP298, LYS347, and GLU349, with interaction arising from hydrogen bonding (LEU112, THR210, ILE252, ILE257, ARG270, GLY271, and HIS272), halogen bonds (GLU72, GLN109, LEI112, GLY113, ASN253, ARH270, and GLU271), polar bonds (LYS79, SER206, THR210, SER251, ASN253, TRP298, LYS349, and GLU349), hydrophobic deliberations (LEU73, LEU112, CYS115, ILE254, ALA262, VAL269, HIS272, ALA273, and TRP298) and cation–π interaction (TRP and TRP298) (Figure 5). The hydrogen bond length in the case of calpain–PD151746 ranged between 2.78 and 3.48, displaying both long- and short-range H-bonding interactions. Hence, calpain–PD150606 showed better hydrogen bonding, stabilized intermolecular energy components, and a larger binding pocket, corroborating the binding energy and inhibition constant values.

### 2.5. MDL-28170 and SJA6017

**Figure 6:**
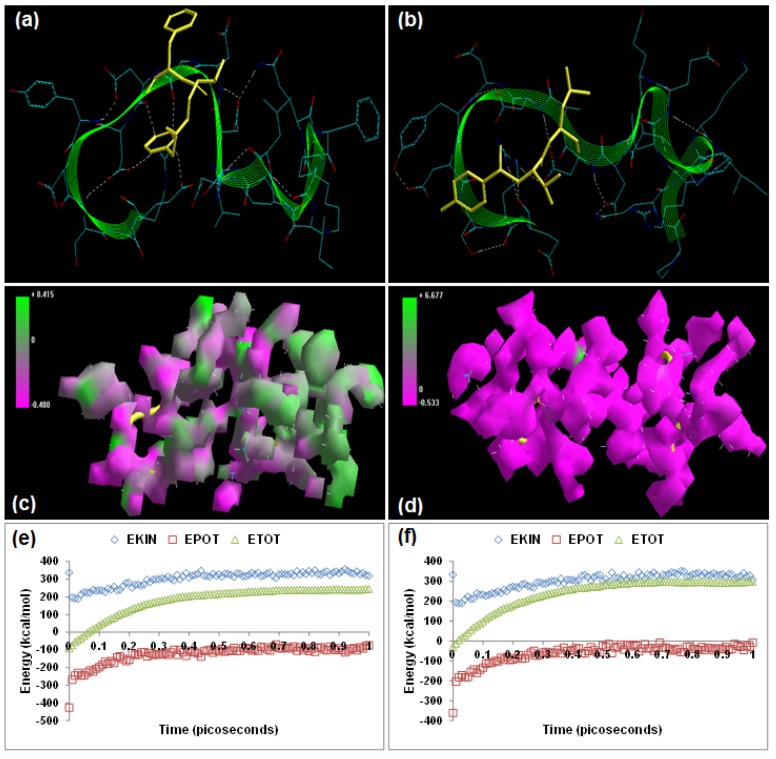

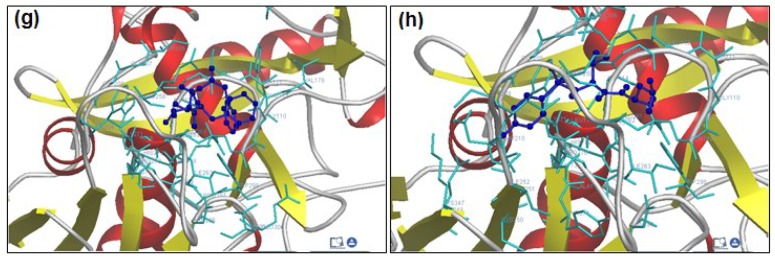
Visualization of the geometrical preferences of the calpain molecule (green secondary structure) in complexation with ligand molecules (yellow tube rendering). (**a**) MDL-28170 and (**b**) SJA6017 after molecular mechanics simulations. 3D-mapped isosurface plot representing the electrostatic potential for (**c**) calpain–MDL-28170 and (**d**) calpain–SJA6017. The kinetic energy (EKIN), potential energy (EPOT), and total energy (ETOT) values for **(e**) calpain–MDL-28170 and (**f**) calpain–SJA6017, calculated by molecular dynamic simulations in vacuum. The most favorable poses of (**g**) MDL-28170 and (**h**) SJA6017 within calpain were obtained via molecular docking.

MDL-28170 ([(1*S*)-2-methyl-1-{[(2*S*)-1-oxo-3-phenylpropan-2-yl]carbamoyl}propyl]amino 2-phenylacetate) and SJA6017 ((2*S*)-2-[(4-fluorobenzene)sulfonamido]-3-methyl-N-[(2*R*)-4-methyl-1-oxopentan-2-yl]butanamide) have been reported to have calpain inhibition capability. Yu and Geddes, 2007, concluded that the combined intravenous and daily intraperitoneal administration of MDL-28170 resulted in “significant improvement in both functional and pathological outcome measures” in a rat SCI model [25]. Akdemir and co-workers, 2008, tested the therapeutic efficacy of SJA6017 in a rat spinal cord injury model and concluded that “treatment with SJA6017 reduces apoptotic cell death, preserves spinal cord tissue and improves functional outcome” [26]. Energetic calculations following MM simulations revealed that MDL-28170 (ΔE_Total_ = −54.56 kcal/mol) conferred better interaction and complexation with calpain than SJA6017 (ΔE_Total_ = −17.023 kcal/mol), which is in agreement with the reported inhibitor profile of MDL-28170 [calpain I (K_i_ = 0.01 μM) and calpain II (K_i_ = 0.01 μM)] and SJA6017 [calpain I (K_i_ = 0.022 μM), calpain II (K_i_ = 0.049 μM)] ([17], Table 1). Interestingly, all the bonding (bond length, bond angle, and dihedral angle) and non-bonding (van der Waals force, H-bonding, and electrostatic interaction) energies demonstrated similar stabilization pattern in the case of calpain–MDL-28170 and calpain–SJA6017. The presence of sulfonamido and fluorobenzene functionalities—presenting higher molecular bulkiness to SJA6017—contributed significantly to the comparative destabilization of energy components in the calpain–SJA6017 protein–inhibitor complex (*V_θ_* = 71.023 kcal/mol). The electrostatic component—the major contributor to the calpain–calpain inhibitor geometrical stabilization—of the calpain–MDL-28170 (−449.013 kcal/mol) was significantly more stabilized than that of calpain–SJA6017 (−441.937 kcal/mol). The 3D-mapped isosurface plots of calpain–MDL-28170 (−0.480 kcal/mol < V < +0.415 kcal/mol) and calpain–SJA6017 (−0.533 kcal/mol < V < +6.677 kcal/mol) depicted major electrostatic surface with very strong positive and negative potentials, respectively (Figure 6). Correspondingly, the MD simulations demonstrated better energetic stabilization in the case of demonstrated calpain–MDL-28170 (−52.664 kcal/mol) than calpain–SJA6017 (−14.572 kcal/mol) with both component kinetic and potential energies contributing to the final geometrical and energetic equilibrium (Table 2).

MDL-28170 and SJA6017 displayed –ve free energy of binding with the proteolytic site of calpain for all binding modes tested (Table 3). As observed in MM and MD simulations, MDL-28170 (ΔE_binding_ = −5.03 kcal/mol) demonstrated better inhibition of calpain, with a Ki value of 204.69 μM as compared to SJA6017 (ΔE_binding_ = −3.59 kcal/mol), which showed a Ki value of 254.11 μM. All the intermolecular energy components—van der Waals + H-bond + desolvation + electrostatic—was more stabilized in the case of the calpain–MDL-28170 complex, which also had a larger interaction surface, producing a better geometrical fit. The binding pocket of calpain–MDL-28170 was lined by GLU72, GLN109, CYS115, TRP116, SER206, GLY208, SER251, ASN253, ILE254, ARG258, ALA262, VAL269, GLY271, HIS272, ALA273, and TRP298, with hydrogen bonding interactions (GLY271, GLY208, and CYS115), polar bonds (HIS272, GLN109, TRP116, and TRP298), hydrophobic involvements (HIS272, VAL269, ALA262, CYS115, TRP116, ILE254, ALA273, and TRP298), and π–π interactions (HIS272 and TRP298) contributing to the binding energy (Figure 6). The hydrogen bonding length in the calpain–MDL-28170 molecular complex ranged between 2.80 and 3.44. In the case of calpain–SJA6017, the ligand–protein binding included GLU72, LYS79, GLN109, GLY110, ALA111, LEU112, GLY113, CYS115, TRP116, GLU203, SER206, THR210, SER251, ASN253, ILE254, ARG258, ALA262, VAL269, GLY271, HIS272, ALA273, TRP298, LYS347, and GLU349, with interaction arising from hydrogen bonding (CYS115, GLY271, and TRP298), polar bonds (GLN109, HIS272, LYS347, and TRP298), hydrophobic deliberations (CYS115, ILE254, HIS272, TRP298, ALA262, TRP116, and ALA273), halogen bonds (GLU72, GLN109, GLY110, LEU112, GLY113, SER106, SER251, and GLY271) and cation–π interaction (TRP298) (Figure 6). The hydrogen bond length in the case of calpain–SJA6017 was 3.27, displaying shorter range H-bonding interactions. In conclusion, calpain–MDL-28170 showed all-component energy stabilization as well as a larger binding pocket in conjugation with low inhibition constant values.

### 2.6. Curcumin

Curcumin ((2-methoxy-4-[(1E,4E,6Z)-7-(3-methoxy-4-oxidophenyl)-5-oxido-3-oxohepta-1,4,6-trien-1-yl]benzen-1-olate)), a polyphenolic phytochemical extracted from the rhizome of *Curcuma longa* Linn., is reported to have neuroprotective and neurotherapeutic properties and is being extensively studied for its potential in spinal cord injury therapeutics [5,27]. The present modeling paradigm was based on the hypothesis that the presence of diketone functionality and the aromatic ring system may contribute extensively to calpain inhibition, as observed in compounds with aldehyde and benzyl moieties. Energetic calculations following MM simulations revealed that curcumin (ΔE_Total_ = −40.213 kcal/mol) produced a geometrically and energetically stable complex with calpain (Table 1). The calpain–curcumin complex was energetically stabilized by all bonding (bond length, bond angle, and torsional strain) and non-bonding (van der Waals forces, H-bonding, and electrostatic interactions) energy terms. Due to the unique symmetrical chemical structure with two aromatic rings on either side of a diketone, calpain–curcumin complexation revealed the best van der Waals interaction profile among the calpain inhibitors tested, with an energy stabilization of ≈−32 kcal/mol (≈75% of energy stabilization). The electrostatic component of the calpain–curcumin complex (−426.87 kcal/mol) was almost equal to the electrostatic energy of calpain (−423.447 kcal/mol), hence displaying complementarity with the calpain molecule. A closer observation of the 3D-mapped isosurface plots of calpain–curcumin and calpain showed high similarity with the various calpain inhibitors detailed above. Additionally, the calpain–curcumin molecular complex (−0.479 kcal/mol < V < +0.838 kcal/mol) presented a strong positive minor electrostatic surface and a major electrostatic surface with strong negative potential, which allowed for a balanced distribution of charge in the calpain molecule. Correspondingly, the MD simulations for calpain–curcumin (−33.527 kcal/mol) demonstrated good energetic stabilization with both component kinetic and potential energies contributing to the geometrical equilibrium (Table 2).

**Figure 7:**
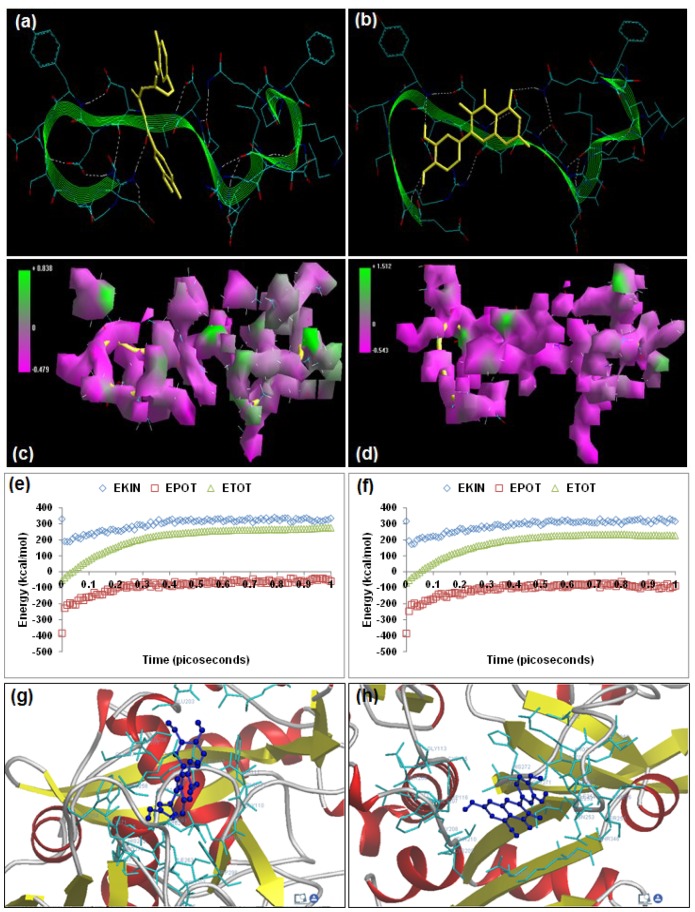
Visualization of the geometrical preferences of the calpain molecule (green secondary structure) in complexation with ligand molecules (yellow tube rendering). (**a**) Curcumin and (**b**) quercetin after molecular mechanics simulations. 3D-mapped isosurface plot representing the electrostatic potential for (**c**) calpain–curcumin and (**d**) calpain–quercetin. The kinetic energy (EKIN), potential energy (EPOT), and total energy (ETOT) values for (**e**) calpain–curcumin and (**f**) calpain–quercetin calculated by molecular dynamic simulations in vacuum. The most favorable poses of (**g**) curcumin and (**h**) quercetin within calpain were obtained via molecular docking.

Curcumin displayed –ve free energy of binding with the proteolytic site of calpain for all binding modes tested (Table 3). Curcumin (ΔE_binding_ = −4.11 kcal/mol) demonstrated inhibition of calpain with a Ki value of 978.21 μM. The intermolecular energy component was stabilized by van der Waals forces, H-bonding, and desolvation energy. The docking studies established two interesting results: (1) the electrostatic energy destabilized the total intermolecular energy and was +ve in magnitude (+0.04 kcal/mol) as compared to –ve magnitude in case of the reference compounds tested, and (2) the interaction surface was narrower than all the reference compounds tested in this study. The binding pocket of calpain–curcumin was lined by GLU72, LYS79, GLN109, CYS115, TRP116, SER206, ASN253, ILE254, ARG258, GLU261, ALA262, VAL269, HIS272, and TRP298, with hydrogen bonding interactions (GLN109 and CYS115), polar bonds (GLU72, HIS272, GLN109, SER206, ARG258, and TRP298), hydrophobic involvements (HIS272, VAL269, ALA262, CYS115, ILE254, and TRP298), π-–π interaction (TRP298), and cation–π interaction (HIS272) contributing to the binding energy (Figure 7). The hydrogen bonding length in the calpain–urcumin molecular complex ranged between 3.02 and 3.26.

### 2.7. Quercetin

Quercetin (2-(3,4-dioxidophenyl)-3,4-dioxo-3,4-dihydro-2H-1-benzopyran-5,7-bis(olate)), a naturally occurring polyphenolic flavonoid, has shown immense potential in SCI therapeutics as an antioxidant and anti-inflammatory agent. This modeling study was inspired by the fact that quercetin consists of a very active chromenone functionality as well as five –OH groups capable of forming H-bonds with biomacromolecules such as calpain [5,28]. The above assumption proved to be true with energetic calculations following MM simulations revealing that quercetin (ΔE_Total_ = −24.825 kcal/mol) produced a geometrically and energetically stable complex with calpain (Table 1). The calpain–quercetin complex was energetically destabilized by the bonding interactions (bond length, bond angle, and torsional strain) and stabilized by non-bonding (van der Waals forces, H-bonding, and electrostatic interactions) energy terms. The destabilization of the calpain–quercetin molecular complex can be attributed to the tricyclic structure of the quercetin experiencing torsional constraints essential for the geometrical fitting of quercetin in the calpain binding pocket. Due to the presence of a hydroxyl group on the entire chemical structure, calpain–quercetin complexation revealed the best H-bonding profile among the calpain inhibitors tested. A closer observation of the geometrical conformation revealed that four out of five -OH groups formed H-bonds with calpain. The van der Waals component of the calpain–quercetin was highly stabilized as compared to the electrostatic energy of calpain (−9.857 kcal/mol) and displayed complementarity with the calpain molecule in terms of electrostatic energy. Interestingly, calpain–quercetin displayed high 3D-mapped isosurface plot similarity with the calpain–curcumin complex. The calpain-quercetin molecular complex (−0.543 kcal/mol < V < +1.512 kcal/mol) presented a strong positive minor electrostatic surface and a major electrostatic surface with strong negative potential, which was in accord with that of the calpain–curcumin complex. Correspondingly, the MD simulations for calpain–quercetin (−24.825 kcal/mol) demonstrated good energetic stabilization, with both component kinetic and potential energies contributing to geometrical equilibrium (Table 2).

Quercetin displayed –ve free energy of binding with the proteolytic site of calpain for all binding modes tested (Table 3). Quercetin (ΔE_binding_ = −4.69 kcal/mol) demonstrated inhibition of calpain with a Ki value of 365.57 μM ≈ three times more potent than curcumin. The intermolecular energy component was stabilized by van der Waals forces, H-bonding, and desolvation energy, as well as the electrostatic energy. The docking studies established two interesting results: (1) the electrostatic energy destabilized the total intermolecular energy and was +ve in magnitude (+0.04 kcal/mol) as compared to –ve magnitude in the case of the reference compounds tested, and (2) the interaction surface was narrower than all the reference compounds tested in this study. In the case of calpain–quercetin, the ligand–protein binding included GLU72, GLN109, CYS115, TRP116, SER206, THR210, SER251, ASN253, ILE254, ARG258, GLU261, ALA262, VAL269, HIS272, ALA273, TRP298, LYS347, and GLU349, with interaction arising from hydrogen bonding (CYS115, SER206, and ILE254), polar bonds (GLU72, THR110, GLN109, HIS272, SER251, ASN253, ARG258, GLU261, TRP298, LYS347, and GLU349), hydrophobic deliberations (CYS115, ILE254, VAL269, and ALA262), π–π interactions (HIS272 and TRP298) and cation–π interactions (TRP116, HIS272, and TRP298) (Figure 7). The hydrogen bond length in the case of calpain–quercetin ranged between 3.32 and 3.51, displaying both long- and short-range H-bonding interactions.

### 2.8. Polyphenols vs. Calpain Inhibitors

**Figure 8:**
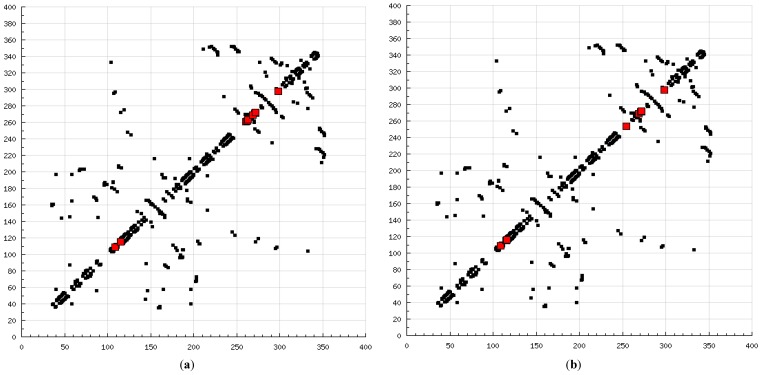

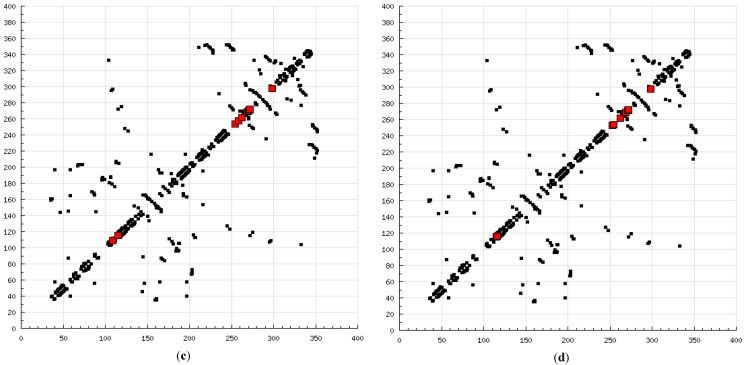

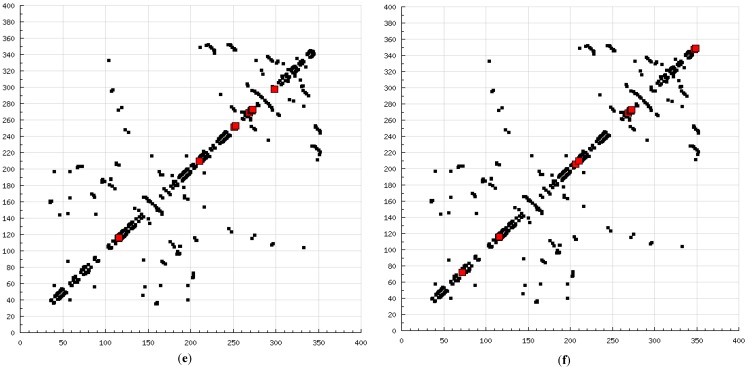

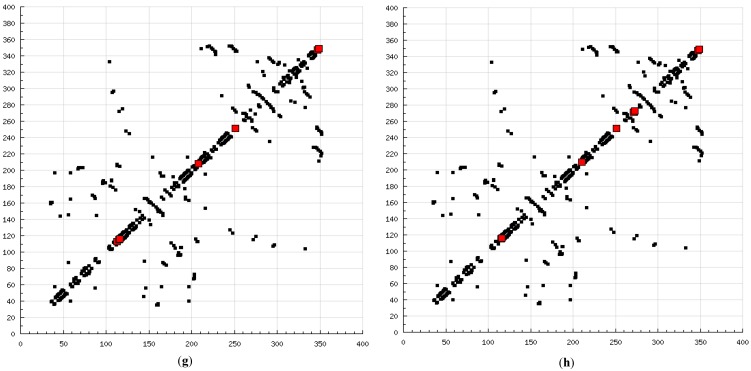

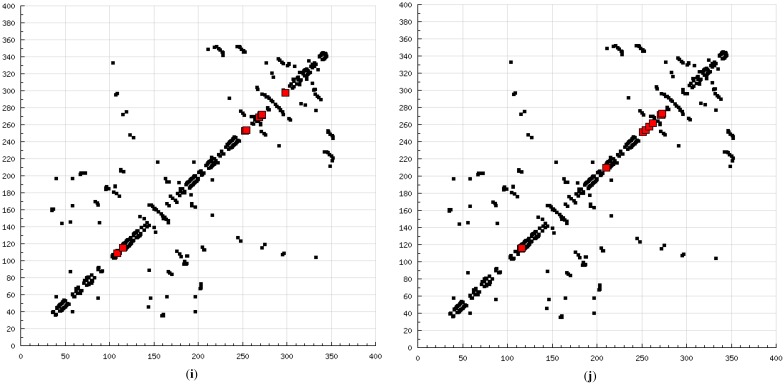

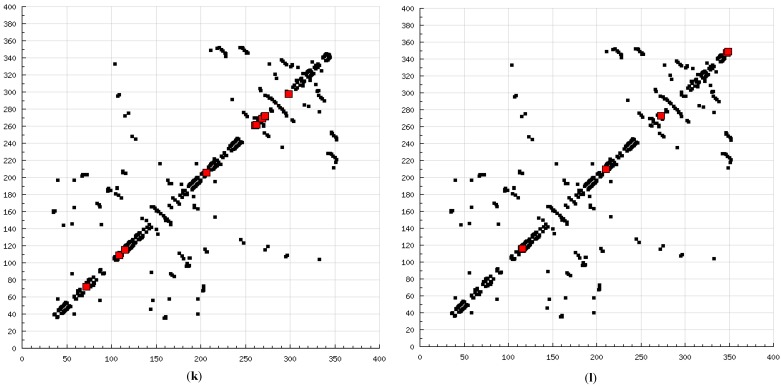
2D HBPlots indicating the differences in the calpain binding modes between coupled inhibitors employed in this study: (**a**) calpain–ALLN; (**b**) calpain–ALLM; (**c**) calpain–AK275; (**d**) calpain–AK295; (**e**) calpain–calpeptin; (**f**) calpain–leupeptin; (**g**) calpain–PD150606; (**h**) calpain–PD151746; (**i**) calpain–MDL-28170; (**j**) calpain–SJA6017; (**k**) calpain–curcumin; and (**l**) calpain–quercetin.

The present modeling simulation study was planned to (1) provide a molecular modeling algorithm, thereby testing if *in silico* simulation can replicate the *in vitro*/*in vivo* inhibition profile of various calpain inhibitors against calpain and (2) ascertain if polyphenols such as curcumin and quercetin can act as calpain inhibitors with efficacy comparable to that of 10 well-known calpain inhibitors. The MM and MD energy stabilization profile of the tested calpain inhibitors can be plotted as follows: SJA6017 < AK275 < AK295 < PD151746 < quercetin < leupeptin < PD150606 < curcumin < ALLN < ALLM < MDL-28170 < calpeptin (Table 1 and Table 2). The above results confirmed that curcumin and quercetin provided better energy stabilization and hence better inhibition than 60% and 40%, respectively, of calpain inhibitors tested. The 3D electrostatic mappings of the calpain–polyphenol molecular complexes displayed complementarity to that of the calpain–ALLM, calpain–leupeptin, calpain–PD150606, and calpain–MDL28170 molecular complexes. The polyphenols complexed with calpain via the formation of strong and weak, minor and major, negative and positive potentials dispersed throughout the isosurface, providing a better geometrical distribution of electrostatic potential across the ligand–protein interface. The calpain–curcumin and calpain–quercetin molecular complexes surpassed the entire calpain–calpain inhibitor mapping in terms of van der Waals interactions and H-bonding due to a highly symmetrical chemical structure and the presence of –OH functionalities, respectively. Furthermore, the stabilization of all energy components in the calpain–curcumin complex can be attributed to the symmetrical structure of curcumin, which was not observed in any of the calpain–calpain inhibitor complexes, confirming the role of chemical symmetry in calpain inhibition. Correspondingly, the established free energy of binding profiles for the tested calpain inhibitors derived from the docking study—AK275 < AK295 < PD151746 < ALLN < PD150606 < curcumin < leupeptin < quercetin < calpeptin < SJA6017 < MDL-28170 < ALLM—conveyed that curcumin and quercetin provided lower established inhibition constant (Ki) values than 50% and 60%, respectively, of calpain inhibitors tested (Table 3). The mutual binding pocket of known calpain inhibitors consisted of GLU72, LEU73, LYS79, GLN109, GLY110, ALA111, LEU112, GLY113, CYS115, TRP116, GLU203, SER206, GLY208, CYS209, THR210, SER251, ILE252, ASN253, ILE254, ILE257, ARG258, GLU261, ALA262, ILE263, VAL269, ARG270, GLY271, HIS272, ALA273, TRP298, LYS347, and GLU349, comprising hydrogen bonding, polar bonds, hydrophobic interactions, halogen bonding, cation–π bonding, and π–π bonding. The polyphenols curcumin and quercetin collectively covered the binding entire pocket from GLU72-GLU349 except ALA111, ARG270, CYS209, GLU203, GLY110, GLY113, GLY208, GLY271, ILE252, ILE257, ILE263, LEU112, and LEU73 and constituted a complete bonding paradigm except for halogen bonding. The above discussion confirmed that polyphenols can potentially inhibit calpain-mediated proteolytic activity after traumatic spinal cord injury, as hypothesized. Figure 8 indicates the differences of the binding modes between coupled inhibitors using HB Plot 2D diagrams and can be an essential tool for targeting protein–inhibitor interactions for the design of compounds with desirable binding properties.

### 2.9. Future Perspective

Spinal Cord Injury (SCI) has been a challenge to treat for several decades, even more so today with the host of experimental treatments available that have many inherent risks for patients. The ideal combination of drugs to be used is not optimized and many clinicians have failed to achieve a therapeutic response due to ignorance of the multiple confounding formulation factors and side effects when dosing a particular steroid. Apart from steroidal interventions, small molecule calpain inhibitors have been employed alone as well as in combination with steroids. However, the non-specificity and low potency of known calpain inhibitors jeopardize their administration after SCI. Polyphenols, if administered in the correct dose, may offer multifaceted therapeutic benefits countering inflammation, free radical-mediated oxidation, and neurodegeneration. In addition, polyphenols such as curcumin and quercetin may offer distinctive advantages via the inhibition of calpain, as described in this study. Individually, these compounds have shown promise but have an unfavorable toxicity profile due to the high doses required and hence there is significant debate over the use of natural compounds such as curcumin and quercetin as alternatives to conventional high-dose steroidal drug therapy. However, a synergistic combination of lower doses of each may prove to be the best option. This paper adds value by hypothetically exploring the magnitude of interactions that exist between polyphenols and calpain. The varied binding pocket of curcumin and quercetin with calpain in addition to wide-ranging energetic modification of calpain’s proteolytic site further confirmed the synergistic potential of curcumin and quercetin. Although the *in silico* results revealed a strong correlation with previously reported *in vitro* results, the estimated inhibition constants of the compounds examined in this study displayed an important deviation from the literature [17,29]. These deviations in the inhibition constant values can be inimitably attributed to (1) the dynamic nature of proteins, (2) non-conservation of binding sites, and (3) random scoring techniques employed in various docking functions [30]. Therefore, the future implications of this study involve (1) generation of a molecular mechanics energy relationship involving multi-ligand–protein interaction studies and multi-ligand docking paradigms; (2) domain-specific (I-VI) as well as overlapping domain specific (I-II, II-III, III-IV, IV-I, II-IV, and II-I) docking analyses of small molecular weight calpain inhibitors in complexation with calpain, taking reference from literature studies; and (3) testing the *in vitro* and *in vivo* calpain inhibition potential of curcumin and quercetin alone and in combination.

## 3. Experimental Section

### 3.1. Static Lattice Atomistic Simulations (Molecular Mechanics)

Molecular Mechanics computations in vacuum, which included the model building of the energy-minimized structures of ligand–calpain complexes, were performed using HyperChem^TM^ 8.0.8 Molecular Modeling software (Hypercube Inc., Gainesville, FL, USA) and ChemBio3D Ultra 11.0 (CambridgeSoft Corp., Cambridge, UK). The calpain 20-mer peptide (PQFKIRLEEVDDADDYDSRE) was constructed using the Sequence Builder Module on HyperChem^TM^ 8.0.8. The structures of the ligands calpain inhibitor I (ALLN), calpain inhibitor II (ALLM), AK275, AK295, calpeptin, leupeptin, PD150606, PD151746, MDL-28170, SJA6017, curcumin, and quercetin were built with pre-defined natural bond angles and charges (Figure 1). The generation of the overall steric energy associated with the energy-minimized structures was initially executed via energy minimization using the MM+ Force Field and the resulting structures were once again energy minimized using the AMBER 3 (Assisted Model Building and Energy Refinements) Force Field. The conformer having the lowest energy was used to create the ligand–protease complexes. To find the most energetically stable geometrical conformation, all possible modes of guest–host interaction were tested. Full geometrical optimizations were performed in vacuum employing the Polak–Ribiere Conjugate Gradient method with the following sequence algorithms: (1) steepest descent method followed by (2) conjugate gradient method to refine the structure. The maximum iteration cycles (termination condition) and the maximum time for the generation of energy minimized, stabilized structures and complexes were based on the target RMS gradient of 0.001 kcal/mol. For computations of energy attributes, the Force Fields were utilized with a distance-dependent dielectric constant scaled by a factor of 1. The 1–4 scale factors followed were electrostatic (0.5) and van der Waals (0.5) [31].

Molecular mechanics energy relationship (MMER), a method for analytico-mathematical representation of potential energy surfaces, was used to provide information about the contributions of valence terms, noncovalent Coulombic terms, and noncovalent van der Waals interactions for ligand–peptide interactions. The MMER model for the potential energy factor in various molecular complexes can be written as:

(1) $${\text{E}}_{\text{molecule}/\text{complex}}=\;V_{\sum }=\;V_{b}+\;V_{\theta }+\;V_{\varphi }+\;V_{ij}+\;V_{hb}+\;V_{el,}$$

where *V_∑_* is related to total steric energy for an optimized structure, *V_b_* corresponds to the bond stretching contributions (reference values were assigned to the bond lengths), *V_θ_* denotes the bond angle contributions (reference values were assigned to bond angles), *V_φ_* represents the torsional contribution arising from deviations from optimum dihedral angles, *V_ij_* incorporates van der Waals interactions due to non-bonded interatomic distances, *V_hb_* symbolizes the hydrogen-bond energy function, and *V_el_* stands for electrostatic energy.

In addition, the total potential energy deviation, Δ*E*_total_, was calculated as the difference between the total potential energy of the complex system and the sum of the potential energies of isolated individual molecules, as follows:

(2) $$\Delta E_{\text{total}\left(\text{A}/\text{B}\right)}=E_{\text{total}\left(\text{A}/\text{B}\right)}-\left(E_{\text{total}\left(\text{A}\right)}+E_{\text{total}(\text{B})}\right)$$

The molecular stability can then be estimated by comparing the total potential energies of the isolated and complexed systems. If the total potential energy of the complex is smaller than the sum of the potential energies of isolated individual molecules in the same conformation, the complexed form is more stable and its formation is favored [32].

### 3.2. Molecular Dynamics Simulations

The ligand–peptide complexes initially minimized by molecular mechanics were then minimized by molecular dynamics for 1.0 ps (time step = 0.001 ps) at 300 K with the Nose−Hoover thermostat. For evaluation of the stability of a simulation and the extent of equilibration and for identification of the interesting low energy conformations, molecular dynamics calculations were averaged and saved as kinetic energy (EKIN), potential energy (EPOT), total energy (ETOT), and temperature (TEMP) Equilibrium was established before recording the measurements, and the instantaneous potential and kinetic energy were monitored to determine when the system reaches equilibrium. Thereafter, the simulation was allowed to run for 1000 time-steps before taking measurements [33].

### 3.3. Molecular Docking Studies

Docking computations were performed using DockingServer. The MMFF94 force field was used for energy minimization of the ligand molecule. Gasteiger partial charges were added to the ligand atoms and nonpolar H-atoms were merged, with rotatable bonds defined. The docking computations were performed using the inhibitor protein model. Essential H-atoms, Kollman united-atom type charges, and solvation parameters were added with the aid of AutoDock tools. Affinity (grid) maps of 20 × 20 × 20 °A grid points and 0.375 °A spacing were generated using the Autogrid program. AutoDock parameter set- and distance-dependent dielectric functions were used for computation of the van der Waals and electrostatic terms, respectively. The docking simulations were performed using a Lamarckian Genetic Algorithm as well as the Solis and Wets local search method. The initial position, orientation, and torsions of the ligand molecules were set randomly. All rotatable torsions were released during docking and each docking experiment was derived from 10 different runs that were set to terminate after a maximum of 25,000 energy evaluations. The population size was set to a value of 150. During the search, a translational step of 0.2 °A and quaternion as well as torsion steps of to the value of 5 were applied [34,35,36].

## 4. Conclusions

This study provided the foremost comprehensive *in silico* evidence across pharmaceutical and medicinal interventions for the potential inhibition of calpain-induced apoptosis. The library of biologically active small molecules such as calpain inhibitor I (ALLN), calpain inhibitor II (ALLM), AK275, AK295, calpeptin, leupeptin, PD150606, PD151746, MDL-28170, and SJA6017 successfully validated the modeling algorithm. The MM, MD, and protein–ligand docking studies effectively quantified the molecular attributes of the protein–ligand(s) interactions in the terms of various pertinent energy attributes and generated preliminary data for protein–ligand sensitivity analysis and interaction studies. The MM and MD energy stabilization profiles displayed the following interaction profiling: SJA6017 < AK275 < AK295 < PD151746 < quercetin < leupeptin < PD150606 < curcumin < ALLN < ALLM < MDL-28170 < calpeptin. The docking analysis demonstrated a calpain inhibition profile in the following order: AK275 < AK295 < PD151746 < ALLN < PD150606 < curcumin < leupeptin < quercetin < calpeptin < SJA6017 < MDL-28170 < ALLM, wherein curcumin interacted with GLU72, LYS79, GLN109, CYS115, TRP116, SER206, ASN253, ILE254, ARG258, GLU261, ALA262, VAL269, HIS272, and TRP298, whereas quercetin exhibited a binding pocket formed by GLU72, GLN109, CYS115, TRP116, SER206, THR210, SER251, ASN253, ILE254, ARG258, GLU261, ALA262, VAL269, HIS272, ALA273, TRP298, LYS347, and GLU349. The modeling paradigm used in this study provided the first ever detailed account of the enzyme inhibition efficacy of calpain inhibitors and the respective calpain–calpain inhibitor molecular complexes’ energetic landscape along with a detailed polyphenol–calpain interaction profile with implications reaching to *in vivo* studies. The whole study can be concluded under the following points:

- Calpain inhibitors may significantly improve biochemical, functional, and behavioral outcomes after SCI by reducing the calpain-mediated proteolysis of cytoskeletal and neurofilament proteins.
- The unique symmetrical chemical structure of curcumin, with two aromatic rings on either side of a diketone, conferred the best van der Waals interaction profile among the calpain inhibitors tested, with an energy stabilization of ≈−32 kcal/mol (≈75% of total energy stabilization).
- The presence of hydroxyl groups on the entire chemical structure accounts for the calpain–quercetin complexation having the best H-bonding profile among the calpain inhibitors tested.
- The experimental inhibitory potential of calpain inhibitors is directly related to the molecular modeling energy relationship (MMER) developed in this study—a first in the field of computational elucidation of inhibitory potential of enzyme inhibitors.
- The varied binding pocket of curcumin and quercetin with calpain, in addition to the wide-ranging energetic modification of calpain’s proteolytic site, confirmed the synergistic potential of curcumin and quercetin.

## References

[1] Banik N.L., Shields D.C., Ray S., Davis B., Matzelle D., Wilford G., Hogan E.L. (1998). Role of Calpain in spinal cord injury: Effects of Calpain and free radical inhibitors. Ann. N. Y. Acad. Sci..

[2] Chai H.-H., Lim D., Jung E., Choi B.-H., Cho Y.-M. (2014). Understanding the Interaction Determinants of CAPN1 Inhibition by CAST4 from Bovines Using Molecular Modeling Techniques. Molecules.

[3] Saatman K.E., Creed J., Raghupathi R. (2010). Calpain as a therapeutic target in traumatic brain injury. Neurotherapeutics.

[4] Ray S.K., Hogan E.L., Banik N.L. (2003). Calpain in the pathophysiology of spinal cord injury: Neuroprotection with Calpain inhibitors. Brain Res. Rev..

[5] Bijak M., Ponczek M.B., Nowak P. (2014). Polyphenol compounds belonging to flavonoids inhibit activity of coagulation factor X. Int. J. Biol. Macromol..

[6] Kumar P., Choonara Y.E., Modi G., Naidoo D., Pillay V. (2014). Cur(Que)min: A neuroactive permutation of Curcumin and Quercetin for treating spinal cord injury. Med. Hypotheses.

[7] Tompa P., Emori Y., Sorimachi H., Suzuki K., Friedrich P. (2001). Domain III of Calpain is a Ca^2+^-Regulated Phospholipid-Binding Domain. Biochem. Biophys. Res. Commun..

[8] Moldoveanu T., Gehring K., Green D.R. (2008). Concerted multi-pronged attack by calpastatin to occlude the catalytic cleft of heterodimeric calpains. Nature.

[9] Hanna R.A., Campbell R.L., Davies P.L. (2008). Calcium-bound structure of calpain and its mechanism of inhibition by calpastatin. Nature.

[10] Boz´oky A., Alexa A., Tompa P., Friedrich P. (2005). Multiple interactions of the “transducer” govern its function in calpain activation by Ca^2+^. Biochem. J..

[11] Croall D.E., McGrody K.S. (1994). Domain Structure of Calpain: Mapping the Binding Site for Calpastatin. Biochemistry.

[12] Qian J., Cuerrier D., Davies P.L., Li Z., Powers J.C., Campbell R.L. (2008). Co-crystal structures of primed side-extending α-ketoamide inhibitors reveal novel calpain-inhibitor aromatic interactions. J. Med. Chem..

[13] Schumacher P.A., Siman R.G., Fehlings M.G. (2000). Pretreatment with Calpain inhibitor CEP-4143 inhibits Calpain I activation and cytoskeletal degradation, improves neurological function, and enhances axonal survival after traumatic spinal cord injury. J. Neurochem..

[14] Fasinu P., Choonara Y.E., Khan R.A., du Toit L.C., Kumar P., Ndesendo V.M.K., Pillay V. (2013). Flavonoids and polymer-derivatives as cyp3a4 inhibitors for improved oral drug bioavailability: An *in vitro* and *in silico* comparative analysis. J. Pharm. Sci..

[15] Saito K., Nixon R.A. (1993). Specificity of calcium-activated neutral proteinase (CANP) inhibitors for human μCANP and mCANP. Neurochem. Res..

[16] Zhu H., Zhang L., Huang X., Davis J.J., Jacob D.A., Teraishi F., Chiao P., Fang B. (2004). Overcoming acquired resistance to TRAIL by chemotherapeutic agents and Calpain Inhibitor I through distinct mechanisms. Mol. Ther..

[17] Pietsch M., Chua K.C.H., Abell A.D. (2010). Calpains: Attractive targets for the development of synthetic inhibitors. Curr. Top. Med. Chem..

[18] James T., Matzelle D., Bartus R., Hogan E.L., Banik N.L. (1998). New inhibitors of Calpain prevent degradation of cytoskeletal and myelin proteins in spinal cord *in vitro*. J. Neurosci. Res..

[19] Colak A., Kaya M., Karaoğlan A., Sağmanligil A., Akdemir O., Sahan E., Celik O. (2009). Calpain inhibitor AK 295 inhibits Calpain-induced apoptosis and improves neurologic function after traumatic spinal cord injury in rats. Neurocirugia (Astur.).

[20] Ray S.K., Wilford G.G., Crosby C.V., Hogan E.L., Banik N.L. (1999). Diverse stimuli induce Calpain overexpression and apoptosis in C6 glioma cells. Brain Res..

[21] Ray S.K., Fidan M., Nowak M.W., Wilford G.G., Hogan E.L., Banik N.L. (2000). Oxidative stress and Ca^2+^ influx upregulate Calpain and induce apoptosis in PC12 cells. Brain Res..

[22] Farkas B., Tantos A., Schlett K., Vilagi I., Friedrich P. (2004). Ischemia-induced increase in long-term potentiation is warded off by specific Calpain inhibitor PD150606. Brain Res..

[23] Chatterjee P.K., Todorovic Z., Sivarajah A., Mota-Filipe H., Brown P.A., Stewart K.N., Mazzon E., Cuzzocrea S., Thiemermann C. (2005). Inhibitors of Calpain activation (PD150606 and E-64) and renal ischemia-reperfusion injury. Biochem. Pharmacol..

[24] Verdaguer E., Alvira D., Jimenez A., Rimbau V., Camins A., Pallas M. (2005). Inhibition of the cdk5/MEF2 pathway is involved in the antiapoptotic properties of calpain inhibitors in cerebellar neuronsInhibition of the cdk5/MEF2 pathway is involved in the antiapoptotic properties of Calpain inhibitors in cerebellar neurons. Br. J. Pharmacol..

[25] Yu C.G., Geddes J.W. (2007). Sustained Calpain inhibition improves locomotor function and tissue sparing following contusive spinal cord injury. Neurochem. Res..

[26] Akdemir O., Uçankale M., Karaoğlan A., Barut S., Sağmanligil A., Bilguvar K., Cirakoğlu B., Sahan E., Colak A. (2008). Therapeutic efficacy of SJA6017, a Calpain inhibitor, in rat spinal cord injury. J. Clin. Neurosci..

[27] Bhullar K.S., Jha A., Youssef D., Rupasinghe H.P.V. (2013). Curcumin and Its Carbocyclic Analogs: Structure-Activity in Relation to Antioxidant and Selected Biological Properties. Molecules.

[28] Katsube M., Kato T., Kitagawa M., Noma H., Fujita H., Kitagawa S. (2008). Calpain-mediated regulation of the distinct signaling pathways and cell migration in human neutrophils. J. Leukoc. Biol..

[29] Moalin M., Strijdonck G.P.F., Beckers M., Hagemen G.J., Borm P.J., Bast A., Haenen G.R.M.M. (2011). A Planar Conformation and the Hydroxyl Groups in the B and C Rings Play a Pivotal Role in the Antioxidant Capacity of Quercetin and Quercetin Derivatives. Molecules.

[30] Kastritis P.L., Bonvin A.M.J.J. (2013). On the binding affinity of macromolecular interactions: Daring to ask why proteins interact?. J. R. Soc. Interface.

[31] Kumar P., Pillay V., Choonara Y.E., Modi G., Naidoo D., du Toit L.C. (2011). *In silico* theoretical molecular modeling for Alzheimer’s disease: The nicotine-Curcumin paradigm in neuroprotection and neurotherapy. Int. J. Mol. Sci..

[32] Choonara Y.E., Pillay V., Ndesendo V.M.K., du Toit L.C., Kumar P., Khan R.A., Murphy C.S., Jarvis D.-L. (2011). Polymeric emulsion and crosslink-mediated synthesis of super-stable nanoparticles as sustained-release anti-tuberculosis drug carriers. Colloid Interface B Biointerface.

[33] Ngwuluka N.C., Choonara Y.E., Kumar P., du Toit L.C., Khan R.A., Pillay V. (2014). A novel pH-responsive interpolyelectrolyte hydrogel complex for the oral delivery of levodopa. I. IPEC modeling and synthesis. J. Biomed. Mater. Res. A.

[34] Bikadi Z., Hazai E. (2009). Application of the PM6 semi-empirical method to modeling proteins enhances docking accuracy of AutoDock. J. Cheminform..

[35] Halgren T.A. (1998). Merck molecular force field. I. Basis, form, scope, parametrization, and performance of MMFF94. J. Comput. Chem..

[36] Morris G.M., Goodsell D.S., Halliday R.S., Huey R., Hart W.E., Belew R.K., Olson A.J. (1998). Automated docking using a Lamarckian genetic algorithm and an empirical binding free energy function. J. Comput. Chem..

